# Genetic and non-genetic factors associated with the phenotype of exceptional longevity & normal cognition

**DOI:** 10.1038/s41598-020-75446-2

**Published:** 2020-11-05

**Authors:** Bin Han, Huashuai Chen, Yao Yao, Xiaomin Liu, Chao Nie, Junxia Min, Yi Zeng, Michael W. Lutz

**Affiliations:** 1 Department of Statistical Science, Duke University, Durham, NC USA; 2 Center for the Study of Aging and Human Development, Medical School of Duke University, Durham, NC USA; 3 Business School of Xiangtan University, Xiangtan, China; 4 BGI Education Center, University of Chinese Academy of Sciences, Shenzhen, China; 5 BGI–Shenzhen, Shenzhen, China; 6 The First Affiliated Hospital, Institute of Translational Medicine, School of Medicine, Zhejiang University, Hangzhou, China; 7 Center for Healthy Aging and Development Studies, National School of Development, Raissun Institute for Advanced Studies, Peking University, Beijing, China; 8 Department of Neurology, Duke University School of Medicine, Durham, NC USA

**Keywords:** Genome informatics, Machine learning, Genome-wide association studies, Cognitive ageing

## Abstract

In this study, we split 2156 individuals from the Chinese Longitudinal Healthy Longevity Survey (CLHLS) data into two groups, establishing a phenotype of exceptional longevity & normal cognition versus cognitive impairment. We conducted a genome-wide association study (GWAS) to identify significant genetic variants and biological pathways that are associated with cognitive impairment and used these results to construct polygenic risk scores. We elucidated the important and robust factors, both genetic and non-genetic, in predicting the phenotype, using several machine learning models. The GWAS identified 28 significant SNPs at *p*-value $< 3 \times 10^{-5}$ significance level and we pinpointed four genes, *ESR1*, *PHB*, *RYR3*, *GRIK2*, that are associated with the phenotype though immunological systems, brain function, metabolic pathways, inflammation and diet in the CLHLS cohort. Using both genetic and non-genetic factors, four machine learning models have close prediction results for the phenotype measured in Area Under the Curve: random forest (0.782), XGBoost (0.781), support vector machine with linear kernel (0.780), and $\ell _2$ penalized logistic regression (0.780). The top four important and congruent features in predicting the phenotype identified by these four models are: polygenic risk score, sex, age, and education.

## Introduction

Cognitive Impairment (CI) is defined as the loss of ability in cognitive functions, such as remembering, learning, and concentrating, which negatively impacts affected individuals’ daily activities^1^. In the stage of mild cognitive impairment (MCI), affected individuals start to experience memory issues without seriously hindering their abilities to execute daily activities. In the stage of severe cognitive impairment, which is referred as dementia^2^, individuals tend to lose basic functionalities of comprehending, memorizing, or even talking and writing. Many diseases are associated with the development of CI, such as Alzheimer’s Disease (AD), Vascular Dementia, Parkinson’s Disease (PD), Progressive Supranuclear Palsy, and Lewy Body Disease^3^.

Aging is one of the major risk factors for the development and onset of CI^4,5^. As forecast in the world population aging report^6^, the population aged 60 years or above is expected to grow by 56% between 2015 and 2030. By 2050, that population is projected to be 2.1 billion globally. The increasing aging population size, with correspondingly increasing prevalence of CI, imposes great burdens at the levels of individuals, families, and communities^7–9^. Thus, it is vitally important to study factors that are associated with CI and to investigate potential therapeutic or lifestyle interventions, for the sake of improving the quality of life or delaying the onset of CI and reducing economic costs.

There have been many previous studies on the pertinent factors associated with cognitive impairment. For example, using discovery and multiple replication cohorts, Davies et al.^5^ identified several significant genetic loci that are associated with CI, such as rs2075650 and rs115566 located in *TOMM40* and rs429358 located in the *APOE* region. Lv et al.^10^ probed the association between the rate of cognitive decline and the mortality rate. They concluded that faster cognitive decline rate is associated with higher mortality rate, specifically among individuals aged between 65-79 years old and cognitively normal individuals, regardless of their initial cognitive abilities. With respect to the analysis on non-genetic factors, Casanova et al.^11^ constructed a random forest model to investigate important predictors for cognitive trajectories, identifying education, age, and gender, as top predictors. Many other studies have also analyzed the genetic and non-genetic contributors to CI in community-based cohorts^4,12,13^.

Nevertheless, there are few studies that analyze the effects from both genetic and non-genetic factors on CI to our knowledge. Consequently, the primary goal of our research is to investigate which factors, both genetic and non-genetic, are significantly associated with cognitive impairment, in contrast to intact cognition in late life. Specifically, we approached the problem from the following two original perspectives:

- We conducted GWAS using the approach of splitting individuals into two groups, considering a phenotype of exceptional longevity & normal cognition. The work by Perez-Gracia et al.^14^ demonstrated that the selection of individuals with exceptionally characteristic, clinically-related phenotypes, referred to as extreme phenotypes, can promote efficiency in identifying important factors at the molecular level. Additionally, Estep^15^ proposed a similar definition of long-living individuals with intact cognition as the extreme phenotype. Consequently, we split the samples into two groups: (1) participants with the exceptional longevity & normal cognition phenotype—aged 90 or above with normal cognition. (2) individuals that were cognitively impaired without age restrictions. The goal here is trying to raise the contrast between the two groups and help identify significant protective effects from the group with exceptional longevity & normal cognition and risk effects from the cognitively impaired group. Note that the definition of exceptional longevity (age ≥ 90 years) can differ in different population and social contexts.
- Machine learning has been widely used for prediction tasks. There has been previous work on applying machine learning techniques to identify the important predictors of cognitive trajectories by Casanova et al.^11^ as mentioned above. In our analysis, we considered several machine learning models to predict CI and identify significant factors using feature importance plots. Different from Casanova et al.’s work where they only worked with random forest models, we considered multiple machine learning models to reduce potential bias from using just one model as model choices have potential impacts on the results. We also introduced genetic effects in the form of Polygenic Risk Scores (PRS) and considered more non-genetic variables.

## Results

### Summary statistics

The non-genetic factors from the CLHLS survey include socioeconomic characteristics, health status, and living habits referring to previous studies^4,10,16^. Specifically, socioeconomic factors include: age, sex, education, occupation, co-residence, and marital status. Living-habit factors include: staple food (major food source), fruits intake, vegetables intake, current smoker, former smoker, current drinker, former drinker, and exercise currently. Health status factors include whether participants have hypertension, diabetes, heart diseases, cardiovascular diseases, and respiratory diseases. Except for age, education, occupation, marital status, and staple food, other factors are binary features taking on yes/no answers. Please refer to Table 1 for summary statistics of non-genetic factors. In terms of our study sample, Table 2 displayed the counts of individuals stratified by sex and age groups (5-year intervals), conditioned on cognitive status. We noticed that there are more female participants than male counterparts and there are more cognitively impaired participants than cognitively normal ones. Almost half of the individuals are within the 100–105 age group. Additionally, we ran univariate tests (T-test and Chi-square tests of Independence) on variables to check if there are differences in the mean values or if there is any association existing between the two cognitive groups and the variables. Please refer to Table 2 in the Supplemental material.

**Table 1:** Summary statistics of non-genetic factors.

Feature	Male (N = 533)	Female (N = 1623)	Total (N = 2156)
Age (years), mean (SD)	100.0 (3.55)	101.7 (3.54)	101.3 (3.62)
Education (years), mean (SD)	2.9 (3.9)	0.3 (1.3)	0.9 (2.49)
**Occupation**			
White-Collar	64 (0.12)	16 (0.01)	80 (0.04)
Other	469 (0.88)	1607 (0.99)	2076 (0.96)
**Marital status**			
Single	450 (0.84)	1598 (0.98)	2048 (0.95)
Partnered	83 (0.16)	25 (0.02)	108 (0.05)
**Staple food**			
Corn	13 (0.02)	54 (0.03)	67 (0.03)
Rice	295 (0.55)	859 (0.53)	1154 (0.53)
Wheat	129 (0.24)	467 (0.28)	596 (0.28)
Other	96 (0.18)	243 (0.15)	339 (0.16)
Co-residence	449 (0.85)	1370 (0.84)	1819 (0.84)
Fruit intake	73 (0.14)	188 (0.12)	261 (0.12)
Vegetables intake	433 (0.81)	1324 (0.82)	1757 (0.81)
Current smoker	93 (0.17)	78 (0.05)	171 (0.08)
Former smoker	221 (0.41)	159 (0.10)	380 (0.18)
Current drinker	103 (0.19)	133 (0.08)	236 (0.11)
Former drinker	205 (0.38)	211 (0.13)	416 (0.19)
Exercise currently	141 (0.26)	185 (0.11)	326 (0.15)
Hypertension	88 (0.17)	286 (0.18)	374 (0.17)
Diabetes	6 (0.01)	12 (0.01)	18 (0.01)
Heart	53 (0.1)	131 (0.08)	184 (0.09)
Cardiovascular disease	29 (0.05)	79 (0.05)	108 (0.05)
Respiratory	77 (0.14)	140 (0.09)	217 (0.10)

Data are provided as count (percentage), unless specified in the feature column. From variable “Co-residence” to “Respiratory”, all the features take binary values of either Yes or No. Their count values sum individuals with Yes response.

**Table 2:** Study sample stratified by age groups and sex, conditioned on cognitive status.

Age groups	Cognitively impaired		Cognitively normal	
	Female	Male	Female	Male
90–95	9	6	12	21
95–100	191	78	156	122
100–105	615	115	316	142
105–110	189	26	95	18
110+	26	2	14	3

The age groups are inclusive on the right bounds and exclusive on the left bounds.

### GWAS result

We present the set of significant SNPs in Table 3 using $3 \times 10^{-5}$ as the p-value threshold. The table contains 28 significant and independent SNPs associated with CI, with 14 SNPs having odds ratios greater than one and 14 less than one. Out of all the significant SNPs, rs13198061 in gene *ESR1*, rs56368572 in *CTNND2*, rs954303 near *RNU4-58P* and rs939432 in gene *RYR3* have p-values less than $1\times 10^{-5}$ and odds ratio less than 1, indicating that those SNPs are associated with potential protective effects of preventing cognitive impairment. The SNPs in *ESR1* and *RYR3* are of specific interest as they have been extensively studied and found to be involved with immunologic processes and brain functions, which are previously reported to be associated with cognitive impairment and decline. Regarding to the significant SNPs that have odds ratios greater than 1, indicating that they are related with progressive effects towards cognitive impairment, we found out that rs935129 in *RP11-81K2.1* and *PHB*, rs6726046 in *DGKD* and *AC0129221.4*, and rs13028996 in *SAG* are significant at the level less than $1\times 10^{-5}$. We also completed the GWAS including years of education as a covariate and report the results in Table 1 in the Supplemental Material.

**Table 3:** Information of significant SNPs from GWAS using $3\times 10^{-5}$ as the *p*-value threshold.

SNP	Chr.	Position	Nearest gene	A1	A2	MAF	*p*	Odds ratio	Lower-95CI	Upper-95CI
$${\mathbf{Odds} }~{\mathbf{ratio }} < {\mathbf{1}}$$										
rs13198061	6	152,306,894	ESR1*	T	C	0.051	$1.4 \times 10^{-6}$	0.49	0.37	0.66
rs939432	15	33,986,294	RYR3*	C	A	0.274	$2.1 \times 10^{-6}$	0.71	0.61	0.82
rs954303	16	59,581,776	RNU4-58P (7606)	A	G	0.155	$2.2 \times 10^{-6}$	0.66	0.55	0.78
rs56368572	5	11,300,912	CTNND2*	T	C	0.094	$8.7 \times 10^{-6}$	0.62	0.50	0.77
rs4816332	21	30,201,706	N6AMT1 (42807)	C	T	0.400	$1.0 \times 10^{-5}$	0.74	0.65	0.85
rs1030695	4	130,318,150	RP11-419L4.1 (91973)	T	A	0.299	$1.1 \times 10^{-5}$	0.73	0.64	0.84
rs1293144	20	52,917,208	PFDN4 (72617)	T	G	0.371	$1.3 \times 10^{-5}$	0.75	0.66	0.85
rs62001981	15	25,279,909	RP11-701H24.10* & PWAR6*	T	C	0.196	$1.8 \times 10^{-5}$	0.70	0.60	0.82
rs9404070	6	101,463,320	GRIK2 (383344)	G	A	0.415	$2.0 \times 10^{-5}$	0.76	0.67	0.86
rs76299633	13	40,727,639	LINC00332 (28307)	G	A	0.119	$2.0 \times 10^{-5}$	0.66	0.55	0.80
rs9676032	18	48,297,450	MRO (27124)	T	A	0.131	$2.1 \times 10^{-5}$	0.67	0.56	0.81
rs28673399	4	71,371,765	AMTN (12492)	G	A	0.448	$2.2 \times 10^{-5}$	0.76	0.67	0.86
rs10500293	19	46,431,638	NOVA2 (5354)	G	A	0.440	$2.7 \times 10^{-5}$	0.77	0.68	0.87
rs72627042	3	23,906,287	UBE2E1*	T	C	0.058	$2.8 \times 10^{-5}$	0.55	0.41	0.72
$${\mathbf{Odds} }~{\mathbf{ratio} } > {\mathbf{1}}$$										
rs13028996	2	234,246,225	SAG*	C	T	0.466	$1.1 \times 10^{-6}$	1.38	1.21	1.57
rs6726046	2	234,287,221	DGKD* & AC019221.4*	A	G	0.375	$1.5 \times 10^{-6}$	1.37	1.21	1.56
rs935129	17	47,486,016	RP11-81K2.1* & PHB*	A	G	0.387	$8.3 \times 10^{-6}$	1.35	1.18	1.53
rs2792251	1	164,541,977	PBX1*	G	A	0.136	$1.1 \times 10^{-5}$	1.53	1.27	1.85
rs6547617	2	85,655,402	SH2D6*	A	T	0.406	$1.1 \times 10^{-5}$	1.35	1.18	1.55
rs10037430	5	180,569,007	OR2V2 (12936)	C	T	0.069	$1.2 \times 10^{-5}$	1.93	1.44	2.59
rs7710849	5	82,220,225	RP11-78C3.1 (3287)	T	A	0.052	$1.3 \times 10^{-5}$	2.04	1.48	2.80
rs79669991	22	43,936,861	-	A	G	0.253	$1.5 \times 10^{-5}$	1.39	1.20	1.61
rs2418761	10	107,295,345	RNU6-463P (822)	C	T	0.110	$1.5 \times 10^{-5}$	1.57	1.28	1.93
rs7927292	11	44,730,158	RP11-45A12.2 (10784)	A	C	0.082	$2.0 \times 10^{-5}$	1.71	1.34	2.18
rs741171	16	6,652,854	RP11-420N3.2* & RBFOX1*	G	A	0.206	$2.3 \times 10^{-5}$	1.41	1.21	1.66
rs57164734	11	44,773,258	TSPAN18*	G	C	0.089	$2.3 \times 10^{-5}$	1.66	1.31	2.11
rs2528812	7	22,446,110	STEAP1B (12953)	C	T	0.420	$2.4 \times 10^{-5}$	1.32	1.16	1.50
rs4934715	10	35,364,992	CUL2*	T	G	0.306	$2.8 \times 10^{-5}$	1.35	1.17	1.55

The nearest genes are either the genes that contain the variants (overlapping) or the nearest upstream/downstream gene to the variants. * indicates overlapping gene. Distances to the nearest upstream/downstream genes are listed in the parenthesis, measured in bp distance.

### Polygenic risk score

As mentioned in the introduction, our primary focus is on the identification of statistically significant factors, both genetic and non-genetic, that are associated with CI. However, correction for multiple testing in GWAS might undermine the power of association analysis to identify small effect-size variants that have biological or clinical importance. Additionally, genetically complex diseases typically involve numerous small effect-size genetic factors. Therefore, to select significant SNPs from GWAS and construct PRS, we tested three different *p*-values, from a relatively stringent one to a comparably relaxed one. Then we used PRS to predict cognitive status (binary response variable—impaired or intact cognition) using Area Under the Curve (AUC) as the measurement. The three *p*-values are $1\times 10^{-5}$, $2\times 10^{-5}$, and $3\times 10^{-5}$ and the corresponding number of SNPs are 7, 19, and 28 respectively.

**Figure 1:**
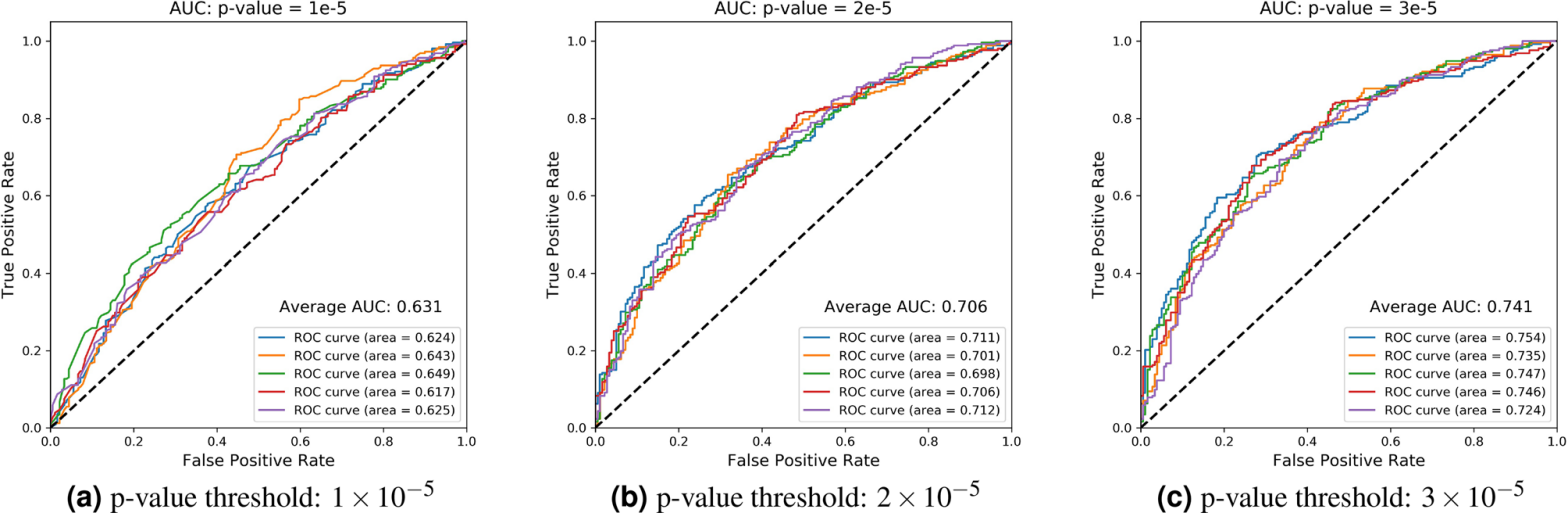
Average AUC from fivefold cross validation using PRS to predict the cognitive impairment. The *p*-value threshold increases from $1\times 10^{-5}$ in panel (**a**) to $2\times 10^{-5}$ in panel (**b**) and to $3\times 10^{-5}$ in panel (**c**).

As shown in Fig. 1, the average AUC from fivefold cross validation noticeably increases as *p*-value threshold increases. Specifically, the PRS from two relatively smaller thresholds ($1\times 10^{-5}$, $2\times 10^{-5}$) have average AUC equal to 0.631 and 0.706 respectively. The PRS from the largest p-value threshold ($3\times 10^{-5}$) has the best performance, with average AUC of 0.742. It demonstrates that an overly stringent *p*-value threshold might overlook some genetic effects, which are small at SNP level but large at aggregated individual level and could enhance the predictive power. Therefore, for the machine learning models, we included the PRS based on SNPs using $3 \times 10^{-5}$ as the threshold, as it has the best performance.

The association between the PRS and the phenotypes in the replication cohort was not significant ($p=0.49$). However, higher scores of the PRS were associated with cognitive impairment relative to the extreme phenotype (age $\ge$ 90 years and no cognitive impairment) (OR = 1.05, 95% CI 0.92–1.20). Female sex was significantly associated with the extreme phenotype relative to cognitive impairment ($p=0.0023$, OR = 0.61, 95% CI 0.45–0.84).

### Machine learning models

Using both genetic and non-genetic factors, four out of five machine learning models we examined—$\ell _2$ penalized logistic regression, support vector machine (SVM) with linear kernel, random forest, and XGBoost have similar performances, with average AUCs from fivefold cross-validation around 0.780. CART has relatively lower prediction performance, with average AUC equal to 0.738. We generated the feature importance plots from the four best performing models and the result are listed in Table 4. Interestingly, all models identified PRS (based on SNPs extracted from GWAS using $3 \times 10^{-5}$ as the *p*-value threshold), education, age, and sex to be important factors, with slightly different orders. It demonstrates that those factors are important and robust in predicting CI. Additionally, simple linear models, including logistic regression and SVM with linear kernel, are consistent on the factor “Vegetables Intake”, while complex models, including random forest and XGBoost, considered intricate interactions among features and agreed on the factors “Staple Food” and “Exercise Currently”.

**Table 4:** Model performances and important features from the best four predictive models.

Model	Performance (AUC)	Top six important features (descending in importance)
$\ell _2$ Penalized Logistic Regression	0.780	PRS, education, age, sex, vegetables intake, former smoker
SVM—linear kernel	0.780	PRS, education, age, sex, co-residence, vegetables intake
Random forest	0.782	PRS, age, education, staple food, sex, exercise currently
XGBoost	0.781	PRS, education, staple food, sex, age, exercise currently

Model performances are almost identical and four importance factors are congruent among the models—PRS, education, age, and sex.

### Pathway analysis

Results of the gene to function pathway analysis are shown in Table 5. Two pathways were significant at the *p* $\le$ 0.05 level after adjustment for multiple comparisons, one is an immunological signature, the other a signature based on a highly conserved transcription factor motif. Two genes are common to these pathways: *ESR1* and *RYR3.*

**Table 5:** Results of the gene to function pathway analysis.

Category	GeneSet	N Genes	N Overlap	P	adjP	Genes	Link
Immunologic signatures	GSE12392_WT_VS_IFNB_KO _CD8A_POS_SPLEEN_DC_DN	200	4	$4.59\times 10^{-6}$	0.022	RYR3:PHB:GRIK2:ESR1	Link1
Transcription factor targets	AACTTT_UNKNOWN	1928	7	$6.09 \times 10^{-5}$	0.037	PBX1:RYR3:RBFOX1:DGKD: UBE2E1:CTNND2:ESR1	Link2

Link 1: http://www.gsea-msigdb.org/gsea/msigdb/cards/GSE12392_WT_VS_IFNB_KO_CD8A_POS_SPLEEN_DC_DN

Link 2: http://www.gsea-msigdb.org/gsea/msigdb/cards/AACTTT_UNKNOWN

“adjP” refers to adjustment for multiple comparisons.

## Discussion

Our study investigated the effects of genetic and non-genetic factors on cognitive impairment. We approached the problem with two novel aspects compared with prior studies. First, we adopted the approach of stratifying individuals with a phenotype of exceptional longevity & normal cognition and contrasted them with cognitively impaired patients using GWAS, aiming to identify significant genetic variants that have progressive effects towards or protective effects from CI. Considering that our data has the world’s largest sample size of Chinese centenarians and considering the historical background of China, this approach would help us identify stronger biological mechanisms. Our study identified numerous significant SNPs in GWAS and pinpointed the corresponding genes that have coherent and inter-connected biological interpretations through immunological systems, brain functions, metabolic pathways, and diets. Second, we used machine learning techniques to predict cognitive impairment using both genetic and non-genetic factors, which have not been well characterized by prior studies. The four best performing models consistently identified four important factors for predicting cognitive impairment: PRS, sex, age, and education. In addition, the two simple models agreed on factor “Vegetables Intake”. The two complex models in addition agreed on two factors: “Exercise Currently” and “Staple Food”, considering convoluted interactive effects among factors.

The PRS is identified as the most important factor from all four models. It is not surprising to see the results because of two reasons. First, genetic pre-disposition affects the onset and severity of CI. Second, as mentioned in section M5, our study considered individuals with the phenotype of exceptional longevity & normal cognition, contrasted with the cognitively impaired individuals, which could potentially identify moderate or strong genetic effects. Age is expected to be significant as ageing is strongly associated with CI. Although the PRS was not significantly associated with the phenotype in the replication cohort, the direction of the effect indicated that this score reflects an increased risk for CI as described in the analysis of the CLHLS data. The effect size of the PRS was also consistent with the effect sizes reported for Alzheimer’s disease genetic associations^17,18^. Replication in an independent daatset is critical for genetic studies including development and testing of PRS. The CLHLS is a unique resource in terms of the number of centenarians and long-lived individuals which makes identification of a suitable replication cohort challenging. The advantages of the ROSMAP cohort for replication are the long follow up peroid with repeated cognitive testing, up to 24 years (mean = 5.6 years, SD = 4.9), consistent definition of the phenotype with the CLHLS study and the large number of individuals with the extreme phenotype (579). Reasons for lack of replication of the PRS association with the phenotype are likely associated with the lack of power to detect a statistically-significant effect. However, it is also possible that adding additional SNPs to the PRS would improve the prediction performance, especially for coverage of individuals with different genetic ancestry. Replication in other cohorts to confirm the usage of the PRS to predict CI relative to aging to 90+ years with intact cognition is warranted.

Prior studies have extensively and consistently shown that sex is an important factor related with longevity, cognitive impairment/decline, and the development of Alzheimer Disease. Au et al.^19^ conducted meta-analysis including 56 studies and showed that there was a higher prevalence, but not incidence, of non-amnestic MCI among women than among men. A similar conclusion has been arrived by Li and Singh^20^: elderly women displayed faster rate of cognitive decline and more severe cognitive deterioration than elderly men. Additionally, closely related with our study population, An et al.^21^ inspected the cognitive patterns among the middle-aged and elder Chinese people, specifically considering sex differentiation. They reported that females tend to have verbal memory advantages over their male counterparts independent of age. On the other hand, males tend to have more intact cognition in general, with better functions in attention, execution, and processing speed. The disparities in cognition exhibited between the two sex groups have not been fully disclosed, but are considered to be the aftermath of differences in years of educations among that generation of the elderly Chinese population^19,21^.

Several studies have shown that education is an important factor in affecting the development of CI^4,12,22^. These studies consistently concluded that higher education (or longer years in education) helps maintain the cognitive functions through cognitive practice, thus reducing the risks of developing CI. Additionally, education has also been extensively studied and widely accepted^23–25^ as an important factor associated with Alzheimer Disease (AD). Similar to the conclusion in CI literature, higher educational levels are associated with lower risk of AD.

As shown in Table 4, simple linear models agreed on “Vegetables Intake” as an important factor. In addition, random forest and XGBoost identified “Staple Food” and “Exercise Currently” as important factors, considering intricate interactions among factors. “Vegetables” and “Staple Food” can be related with an individual's type of diet. The finding that the staple food is an important factor is interesting, specifically considering the environment of China where our study is based. China is generally divided into Southern and Northern areas according to some geographical division. Consequently, the living habits, including the main source of food, are usually different between the two regions, which might have an impact on the starch and diet-related biology. In general, there are some studies that have shown that cognitive decline/cognitive aging is associated with the individual’s diet. For example, two studies^26,27^ have shown that Dietary Approach to Stop Hypertension (DASH) and Mediterranean diet is associated with slower rates of cognitive aging. The study by Samieri et al.^28^ concluded that diets involving fish in-take may slow down the rate of cognitive aging as well. Another study^29^ demonstrated that different types of nutrition in the diet can modify the potential risk of developing cognitive impairment in the future.

In addition to the general studies of the relationship between diet and cognitive decline, there are some specific works that investigate the association between cognitive abilities and diet among Chinese elderly people, which is move relevant to our study. Two research studies^30,31^ have consistently shown that a healthier diet, such as diets with more nuts, vegetables, and fruits, could help decrease the risks of cognitive impairment. Similarly, the study by Wang et al.^32^ arrived at the conclusion that Chinese diets that lack of legume and animal oil might increase the prevalence of mild cognitive impairment. Additionally, it is recognized that Chinese diets are abundant in carbohydrates, mainly starch and sucrose from sources such as rice. The study by Qin et al.^33^ has shown that a wheat-based diverse diet that consists of similar components as the Mediterranean diet could slower the rate of cognitive decline.

Active physical exercise is also found to be important in predicting CI from the two complex models. However, prior human and animal studies investigating the relationship between physical exercise and cognitive functions are not always consistent^34^. Some displayed strong positive associations while other showed minimum or no relationships. For example, Baker et al. studied the effects of aerobic exercises on cognition using randomized and clinically controlled trials. They demonstrated the sex-specific effects that aerobic exercises help improve executive control process for older women^35^. The study from Laurin et al. also showed that physical exercises are associated with lower risk of CI, dementia and Alzheimer Disease, compared with individuals with no exercises^36^. Similarly, Geda et al. conducted a population-based case-control study and concluded that moderate exercise in midlife or latelife, no matter what frequency of the exercise, is associated with reduction in odds of developing mild cognitive impairment (MCI)^37^. However, there are studies that did not identify significant relationships between CI and physical exercise. Young et al. concluded that with their collected randomized controlled trials (RCTs), there is no evidence of cognitive benefits among cognitively healthy elderly people from cardiorespiratory exercises^38^.

Four genes identified in the pathway analysis (*ESR1*, *PHB*, *RYR3*, *GRIK2*) constitute part of an immunologic signature in the MSigDB database^39^ that was identified as significantly associated with the cognitive impairment phenotype. Both the individual genes that comprise this signature, and innate and adaptive immunity as biological processes have been associated with cognitive impairment and cognitive aging^40–46^. Polymorphisms in estrogen receptor genes (*ESR1* and *ESR2*) have been associated with risk of developing cognitive impairment and in turn may play a role in cognitive aging^42,47^. These polymorphisms have been demonstrated to have an impact in both men^42^ and women^42,48^. Moreover, interactions between *ESR1* and the *APOE* gene for AD risk have also been reported^49,50^. Some of the *ESR1* polymorphisms associated with Alzheimer’s disease and mild cognitive impairment are low-frequency (MAF${<}2\%$)^51^. The specific *ESR1* SNP detected as associated with cognitive impairment in our study is not in linkage disequilibrium with the SNPs reported in Yaffe et al. (r2 $\le$ 0.014) and are greater than 100 kB distant from these SNPs, therefore likely represent a different association signal. Genetic alterations in the estrogen metabolic pathway have been reported to be associated with risk of Alzheimer’s Disease in a study of a southern Chinese population^52^ and association of *ESR1* with one-year cognitive decline in healthy oldest-old individuals has also been cited^53^.

The *RYR3* gene codes for the ryanodine receptor that functions to release calcium from intracellular storage for use in cellular signaling and biochemical processes. *RYR3* is an isoform of the ryanodine receptor that is expressed in specific regions of mammalian brain that are involved in the development of cognitive dysfunction and Alzheimer’s disease, e.g. the hippocampus^54^. Sustained calcium dysregulation contributes to neurodegeneration and cognitive impairment^55,56^ and ryanodine receptor levels change during the lifespan^57,58^ and are altered in mild cognitive impairment and Alzheimer’s disease^59^.

The other two genes in the signature, *GRIK2* and *PHB* have literature support for roles in cognitive aging and longevity. *GRIK2* belongs to the kainate family of glutamate receptors that function as ligand-activated ion channels. This gene is highly expressed in brain and mutations in the gene have been associated with cortical development, autism and schizophrenia^60–62^. Interestingly, a large (6036 cases) genome-wide association study identified SNPs near *GRIK2* as showing suggestive levels of association with longevity (OR = 1.2, $p=5.09e^{-8}$)^63^. *PHB*, or prohibitin, is characterized as playing a role in human cellular senescence and tumor suppression and in model organisms, as a modulator of longevity^64^. Prohibitins modulate mitochondrial fusion and have a role in forming protein and lipid scaffolds^65,66^. *Caenorhabditis elegans* studies have shown that prohibitins moderate fat metabolism and energy production and in turn influence aging^64^. In a mouse model, mutations in *PHB2* (one of the two homologous *PHB* proteins) triggered massive neurodegeneration with accumulation of abnormal mitochondria and hyperphosphorylated tau^67^. Interestingly, in a *C. elegans* model, mutations in *PHB* were strongly associated with genotype-dependent responses to dietary restriction^68^.

The immunologic signature based on gene set enrichment for the genes in the predictive risk score represents a small subset (4 out of 200) of genes that are downregulated in CD8A+ splenic dendritic cells in a mouse model where interferon beta 1 (*IFNB1*) is knocked out^69^. *IFNB1*, along with other type 1 interferons link the innate and adaptive arms of the immune system. The involvement of genes representing several metabolic and signaling functions and, specifically, the role for dendritic cells that comprise the biochemical basis for the immunologic signature, contribute to a possible role for association with the cognitive aging phenotype utilized in our study. Dendritic cells capture antigens, which are transported to the lymphatic system. Mechanistically, identification of this signature in the context of our study, that examined cognitive resilience in nonagenarians and centenarians, is of interest because of the likely involvement of the immune system and immune response to exogenous antigens. In the mouse model associated with the signature identified in our study, the absence of *IFNB1* constitutes an “exceptional phenotype” for a model where a critical factor needed for T cell stimulation is removed. Over the course of a lifetime, an individual is exposed to numerous bacterial, viral and parasitic infections. Of particular relevance to our study from the perspective of examining environmental and genetic factors that contribute to long-term cognitive resilience, low but constitutive production of *IFNB1* was shown to be necessary to maintain dendritic cells in a state that is responsive to antigens associated with these infections^69^. Dendritic cells that can capture and process antigens under noninflammatory conditions are considered to acquire tolerogenic properties (e.g. induction of tolerance) which may have strong relevance for understanding the genetic backgrounds associated with cognitive resilience^69^. The overall immunologic gene signature is complex with components including members of gene families of transcription factors, cell differentiation markers and homeodomain proteins. Biochemical linkages between the 4 genes in the signature identified in our study likely represent several underlying physiological processes involved in immune function and not a single biochemical pathway that involves signaling between these genes.

The design of our study is to compare individuals who live to an advanced age, 90+ years with individuals who develop cognitive impairment in middle to late life. Cognitive impairment can result from several pathological processes including Alzheimer’s disease, vascular dementia, cerebrovascular disease, Lewy body dementia, frontotemporal dementia and mixed dementia. The CLHLS survey does not include clinical diagnoses that differentiate these causes and therefore cognitive impairment is equivalent to all cause dementia for the purposes of our study. Alzheimer’s disease pathology is likely a major cause of many of the dementia cases in our study, accounting for 60 to 80% of dementia cases^70^.

Genome-wide association studies (GWAS) and development of predictive risk scores for AD are active areas of research. The study by Kunkel et al.^71^ identified 25 genetic loci associated with AD with many loci supported by earlier GWAS studies^72,73^. Mapping genes to the SNPs identified in the GWAS study enumerated a list of genes that have been investigated through pathway analysis, fine mapping, gene-based association analysis. AD risk-genes include *APOE*, *PICALM*, *BIN1*, *CR1* and *TREM2*; these risk genes and loci are often presented as a Manhattan plot^71^. Our study did not identify any of the AD risk genes as associated with the cognitive phenotype, however the phenotypes in the studies are different in terms of cognitive impairment instead of diagnosed AD and a comparison group of individuals resistant to cognitive impairment until late life ($> 90$ years) in comparison to cognitively normal individuals, typically aged 50–80. Interestingly, there was considerable overlap between the biochemical pathways identified in our study and the biochemical pathways associated with AD, notably immune system and inflammation^71,74–77^. Calcium regulation is biological process involved with AD^78–80^ and the *RYR3* gene identified in our study is a key molecule involved in this process^81^. Lipid metabolism is a key pathway identified through pathway analysis of AD GWAS^71^ and *ESR1* polymorphisms have been shown to impact this pathway^82^, including lipid metabolism in the brain^83,84^.

The predictive risk score (PRS) developed for this study predicted cognitive impairment with AUCs approximately 0.74. This level is similar to a risk score used to predict MCI vs. controls that had an AUC of 0.67. For pathologically-confirmed AD cases, as compared with clinical diagnosis, a PRS score demonstrated an AUC of 0.84^85^, among the best performance for predictive biomarkers for AD that included imaging, biofluid and cerebrospinal fluid based measures.

Studying genetic and non-genetic factors that contribute to the absence or delayed onset of cognitive impairment in resilient individuals may inform lifestyle and therapeutic opportunities for intervention. The biological mechanisms involved in cognitive resilience are complex and our study supports roles for several key metabolic pathways and genes. Strengths of this study include a large cohort comprised of elderly individuals where a substantial proportion demonstrated resilience to cognitive impairment with ages greater than 90 years and consideration of both genetic and environmental factors. Our study also has some limitations. Although the CLHLS cohort contains a large number of centenarians, the sample size is still relatively small for GWAS. Moreover, replication in an independent cohort would be essential to increase the statistical rigor of the genetic association results. Unfortunately, there is no equivalent dataset of individuals of similar ancestry where the dataset is enriched for centenarians and long-lived individuals, essential for our study design. In recognition of these limitations, we have interpreted the biological and gene-specific results in context of prior studies and also used a polygenic risk score to provide a single composite measure of risk from numerous small effect size genetic variants. These steps improve the likelihood for replication. The study represents one of few studies conducted in Han Chinese in contrast to numerous genetic studies of individuals of European ancestry. Future studies are needed to examine whether the results of our study will translate to individuals of other ancestries.

## Methods

### M1. Chinese longitudinal healthy longevity survey (CLHLS)

We worked with the Chinese Longitudinal Healthy Longevity Survey (CLHLS) Series, which are publicly available from National Archive of Computerized Data on Aging (NACDA)^86^. The survey data provides information about physical and mental health status, socioeconomic status, demographics of participants aged 65 and above from 22 provinces in China. The first wave of survey started in 1998, followed with six more waves conducted in 2000, 2002, 2005, 2008–2009, 2011–2012, and 2014. Genotype data is available for 4477 participants with coverage over 7,000,000 SNPs after imputation.

The CLHLS has the world’s largest sample size of centenarians to date^87^. Unlike the population structure of western countries, consisting of people with different ancestries, the population structure in China is comparably homogeneous, with fewer immigrants from other parts of the world. Additionally, even though China has 55 minority groups, Han Chinese (the Han group) accounts for 92% of the total population. To obtain homogeneous genetic information, the surveys only included Han Chinese. A study by Xu and Jin^88^ has shown that the average differences in genetics among European population ($F_{st} = 0.009$) was much higher than that among the Han Chinese population ($F_{st} = 0.002$), based on Human Genome Diversity Panel data. Consequently, the nature of the CLHLS data is less likely to be affected by the population stratification than western cohorts.

**Figure 2:**
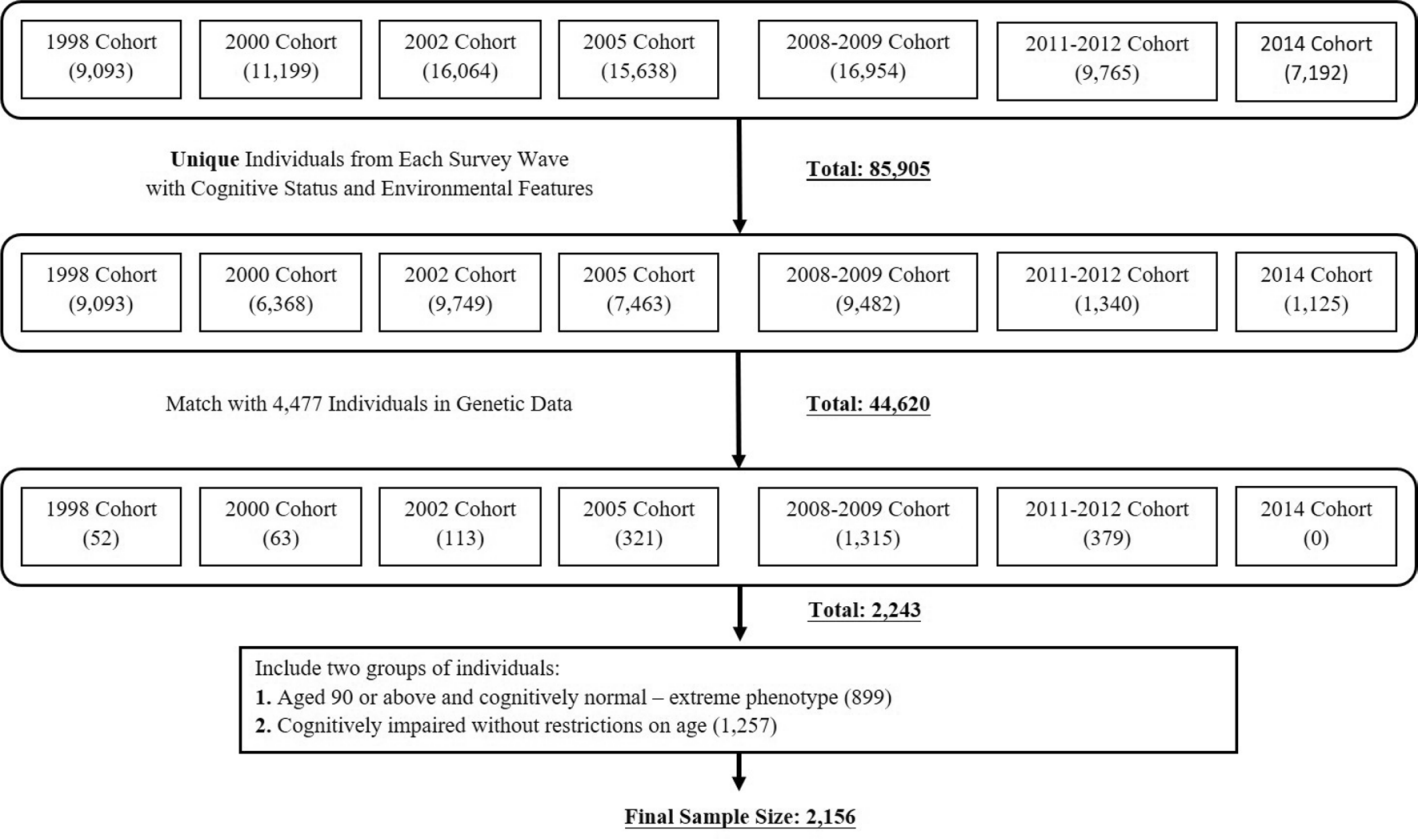
Composition of the study sample. The numbers in the parenthesis are the sample size for each survey cohort.

We utilized the information from the last survey that each individual participated in before he/she was lost-to-follow-up or deceased. For example, if the participant was deceased in the 2005 survey wave, then we used the survey information from 2002 wave. As the final step, we matched participants from the survey with the 4477 individuals who have available genotypes. Since not all participants in the survey have DNA information and not all individuals with genotypes were engaged in the survey, out of 4477 individuals, we ended up having 2243 individuals as our sample. Notice that the final sample has 2165 individuals due to the selection of individuals with a phenotype of exceptional longevity & normal cognition. The composition of the study sample is shown in Fig. 2.

### M2. Genetic data and quality controls

The participants were genotyped using the Illumina HumanOmniZhongHua-8 BeadChips, which was built by selecting optimized tag SNP content from the 1000 Genomes Project and the three HapMap phases^87^. To further increase genome coverage, imputation analysis was performed to infer the genotypes of all SNPs (MAF $\ge$ 0.01) using IMPUTE software version 2^89^ and the 1000 Genomes Project integrated phase 1 release as reference panel. SNPs with a quality score (Rsq) of $< 0.9$ were discarded before analysis. Gene dosages from SNPs that were directly genotyped were used when available, otherwise imputed dosages were used. Therefore, missing values were possible for the directly genotyped SNPs. For SNPs with a substantial frequency of missing data, we used a proxy SNP in the PRS as detailed in M8. More details of the genotyping are articulated in the study by Zeng et al.^87^.

Genotype data prior to quality control consists of 4477 individuals and 7,443,066 SNPs. We imposed quality controls on the set of genetic data using PLINK (v1.9)^90^. The maximum per-SNP missing (–geno), Hardy–Weinberg disequilibrium *p*-value (–hwe), and maximum per-person missing (–mind) are set to default values, which are 0.1, 0.0001, and 0.05 respectively. The minimum minor allele frequency (–maf) is set to 0.05. Post quality control, 4,611,702 SNPs were available for analysis. All the subsequent analyses, such as regional stratification using PCA, frequency calculation, and GWAS, are all based on the genetic data that passed the quality controls.

### M3. Non-genetic data imputation and outcome assessment

We used Mini-Mental State Exam (MMSE) score to measure the outcome—individuals’ cognitive functions. The questions used to calculate MMSE in the Chinese version were carefully designed based on international standards^91^. Both internal^91^ and external^92^ assessments have been conducted and ensured the quality and validity of the questionnaire. The maximum MMSE score is 30. If a person scores 18 or above, he/she is scored as cognitively normal; otherwise, cognitively impaired. We chose 18 as the cutoff value based on several prior studies^4,12^ on cognitive impairment. For each participant in the survey, we calculated MMSE from each survey wave that he/she participated in, until the participant was lost-to-follow-up or passed away. If participants were not able to answer the survey questions—except for the question about number of years of education, we treated the answers as “Wrong” with corresponding 0 score.

There are missing values in some non-genetic factors. Count of missing values are listed in the Table 6, from which we could see that the missing rates are generally low. We imputed the missing values using the Multivariate Imputation by Chained Equations (MICE) Package^93^ in R (v3.6.2)^94^. The imputations were done using CART (Classification and Regression Tree) to account for different types of the factors and potentially complex relationships among the factors. The item and individual missing rates were checked, as well as the distribution of imputed values, to ensure the quality of imputation.

**Table 6:** Count of missing values of non-genetic variables.

Variable	Count of missing value (%)
Education	10 (0.44%)
Occupation	126 (5.61%)
Marital status	11 (0.49%)
Co-residence	9 (0.40%)
Staple food	1 (0.04%)
Fruit intake	4 (0.18%)
Vegetables intake	5 (0.22%)
Current smoker	4 (0.18%)
Former smoke	1 (0.04%)
Current drinker	10 (0.45%)
Former drinker	2 (0.09%)
Exercise currently	19 (0.85%)
Hypertension	76 (3.39%)
Diabetes	87 (3.88%)
Heart	71 (3.17%)
Cardiovascular disease	75 (3.34%)
Respiratory	63 (2.81%)

Variables that do not have missing values are not listed.

### M4. Regional stratification using PCA

It is traditionally thought that China has two major regions—Northern and Southern China, based on geographical division, where people maintain different living habits under the influences of climates and natural resources. Empirically, the study by Xu et al.^95^ have examined and showed internal differences between the Han Chinese population in Southern and Northern areas. Zeng et al.^87^ worked with the same CLHLS data sets and confirmed that the top two eigenvectors are sufficient to adjust the population stratification. Therefore, to account for the potential variations in population characteristics from two regions, we conducted Principle Component Analysis (PCA) and extracted the top two eigenvectors corresponding to the first and second largest eigenvalues. The two eigenvectors are further used as covariates in the GWAS.

### M5. Selection of exceptional longevity & normal cognition phenotype

The 2156 individuals from the sample were split into two groups for GWAS. The first group includes individuals that were aged 90 or above, but were still cognitively intact. We designate this group of people as individuals with the phenotype of exceptional longevity & normal cognition. The other group consists of individuals that are cognitively impaired without any age constraint. The term “extreme phenotype” comes from the study by Perez-Graia et al.^14^. They concluded that samples with extreme phenotype have abundant genetic information about risk or protective effects from SNPs. Considering the nature of the CLHLS cohort and the goal of our research, emphasizing participants with the phenotype of exceptional longevity & normal cognition can potentially help us identify biological mechanisms with strong effects on cognition. In contrast with previous GWAS studies on individuals from western countries, the historical events that happened in China in the twentieth century are drastically different, imposing some external effects on participants from CLHLS^91^. Therefore, the long-lived people in China might have enriched genetic backgrounds that interact with the non-genetic factors, such as environmental factors, to provide protective effects on CI, considering the brutal and harsh environments they have suffered from and survived in the past, such as national and civil wars, revolutions, and starvation.

### M6. Genome-wide association study

The GWAS analysis was performed using logistic regression using PLINK(v1.9), conditioning on sex and the two eigenvectors extracted from the PCA. Both genetic variants and environmental factors have been shown to contribute to educational attainment^96^. Studies have also demonstrated that genetic variants linked to education also predict longevity^97^. Since the study design centers on the GWAS identifying genetic factors associated with cognitive impairment while analysis of environmental factors, including educational attainment is considered in the second phase, we included age, sex and population stratification as covariates in the GWAS and not educational attainment. This also removed the potential multiple confounding between cognitive measures, educational attainment and longevity at the level of the GWAS and is consistent with covariate adjustment in a recent large-scale study of genetic loci influencing general cognitive function^98^.

Chagnon et al.^99^ discussed two general approaches of how to choose the *p*-value threshold in order to identify the significant SNPs: (1) If the goal is to identify SNPs with stronger statistical power, then it is suggested that a more stringent *p*-value threshold be selected, which usually gives less than 100 SNPs. (2) On the other hand, if the scientific question is approached as a prediction problem where strong prediction power is desired, we should not ignore less significant SNPs with larger p-values. Aggregating the small contributions from each SNP could enhance the prediction results. Under this circumstance, researchers usually proceed with a relaxed threshold which generates hundreds more SNPs than a stringent *p*-value.

Even though our approach is prediction-based, our primary focus is on the investigation of statistically important genetic and non-genetic factors in affecting CI. Therefore, we used 100 as the checkpoint for the number of significant SNPs from GWAS. We experimented with three *p*-value thresholds—$1 \times 10^{-5}$, $2 \times 10^{-5}$, and $3\times 10^{-5}$, which gave us 15, 41, and 76 significant SNPs respectively. Using thresholds of $4 \times 10^{-5}$ and $5 \times 10^{-5}$ gave us 120 and 170 SNPs correspondingly, which are more than the checkpoint 100 so we stopped at $3 \times 10^{-5}$. Those are preliminary SNPs with potentially high linkage disequilibrium. We used the –clump command in PLINK (default setting on three parameters: –clump-p2 0.01, –clump-r2 0.5, –clump-kb 250) to clump them into smaller subsets of independent SNPs. The numbers of clumped SNPs are 7, 19, and 28 corresponding to the three *p*-values. All the analyses, including constructing the PRS and machine learning models and biological pathway analysis, were based on the clumped sets of SNPs.

### M7. Missing dosage replacement

To account for substantial missing values for SNPs directly genotyped, we replaced the missing values with the dosage values from proxy SNPs using the following approach:

- For those SNPs not in the dbSNP database, such that they do not have any reference number (“rs” number), we use Kaviar (v160204-Public)^100^ software to map the location with the corresponding reference number. We used the hg19(GRCh37) coordinate system.
- For each significant SNP, we looked up proxy SNPs using LDlink (v4.1.0)^101^. The reference panel was the population of Han Chinese in Beijing, China. All the proxy SNPs were checked to be on the same chromosome as the original SNP. Additionally, as discussed by Chagnon et al.^99^, proxy SNPs with $R^2 \ge 0.8$ are considered to be very good proxies. Therefore, we only include proxies with $R^2 \ge 0.8$.
  For each significant SNP, we created two reference panels. The first panel, “all_panel”, contains all the proxy SNPs for each target SNP. Those proxy SNPs are listed in descending order in terms of $R^2$, so that when we replaced missing dosage values, we always checked the proxy with largest $R^2$ first. The second panel, “reverse_panel”, contains proxies that have different minor alleles from the target SNPs, adjusting the minor allele for the proxies accordingly. For instance, if the minor/major allele of the original SNP is A/T, while the proxy SNP has T/A, then we put this proxy SNP into the reverse panel. Note that the “reverse_panel” is a subset of the “all_panel”. When replacing the missing dosage value, if the proxy SNP is in the “reverse_panel”, instead of directly using the dosage value from the proxy SNP, we adjust the dosage to the allele count.
- For each individual and for each significant SNP where the dosage value is missing, we replaced it with the dosage value from the best (highest $R^2$) and available (existing in our genome data) proxy SNP. If the best available proxy is in the “reverse_panel”, then we used (2-dosage value).
- All remaining missing dosage values were set to zero. The underlying assumption for this is that we expect that most of the population have at least one copy of the major allele, which is best estimated by the 0 dosage value.

### M8. PRS construction

PRS can be viewed as a simple model used to predict the risk for genetically complex diseases. PRS is calculated by aggregating SNP-level information from GWAS to “account for the phenotypic variation observed in complex traits, by assuming an additive, non-multiplicative, effect of multiple variants with variable effect sizes” (Ibanez et al. 2019)^102^. This aggregated information has stronger predictive power for complex diseases than individual genetic variants, notably when effect sizes are small to moderate. With the beta coefficient of each significant SNP_*i*_ and the corresponding dosage value for each individual, we constructed a PRS for each individual *j* using the formula:

$${\text {PRS}}_j = \sum _{i=1}^n \beta _i * {\text {dosage}}_{ij}$$

where $\beta _i$ is the beta coefficient for ${\text {SNP}}_i$ and ${\text {dosage}}_{ij}$ is the corresponding number of effect alleles from the individual j. The PRS can be viewed as the summation of genetic effects that each participant possesses, associated with the underlying probability of developing CI. Then, PRS was used as a single predictor to predict the likelihood of CI. We constructed PRS using three different *p*-value thresholds and assessed their performances correspondingly. We applied fivefold cross validation to evaluate the PRS’s performance. The entire data was split into fivefolds. We evaluated the performance of PRS five times for each of the fivefolds, using Area Under the Curve (AUC) and reported the average of the results.

The PRS derived from the CLHLS GWAS analysis was tested for association with the equivalent phenotype in an independent cohort, the Rush ROSMAP (Religious Order Study/Memory and Aging Project). Participants came from two community-based longitudinal cohort studies of diverse participants, the Religious Orders Study (ROS)^18^, the Rush Memory and Aging Project (MAP)^103^. The studies were approved by the Institutional Review Board of Rush University Medical Center. Participants were enrolled without known dementia and each participant signed an informed consent and agreed to annual clinical evaluations. The ROS and MAP participants were predominantly white Americans. Importantly, both studies were conducted by the same team of investigators and share a large common core of testing batteries and uniform structured clinical evaluations. This makes it possible for a combined analysis. The same phenotype definition, based on survival to age 90 years with intact cognition versus cognitive impairment at any age was used, based on the MMSE scores obtained for the participants. Descriptive statistics for the cohort are included in Supplementary Table 3.

### M9. Machine learning models

In addition to PRS, we considered machine learning models to predict the phenotype—binary cognitive status, using both genetic and non-genetic factors and to identify important predictive factors by assessing feature importance. We examined five machine learning models—$\ell _2$ penalized logistic regression, classification and regression tree (CART), support vector machine (SVM), random forest, and boosted decision trees (XGBoost implementation). Five models were utilized so that we were able to compare their prediction performances and evaluate feature importance, which could potentially reduce the bias from simply assessing one model and help us pinpoint important factors that are robust and congruent from different models.

- $\ell _{2}$ *Penalized Logistic Regression* an $\ell _2$ penalty term is applied to the sum of squared coefficients from logistic regression to present over-fitting.
- *Classification and Regression Tree (CART)*^104^: the algorithm constructs binary decision trees by splitting input features on certain values that maximizes information gain at each note. It stops when a criterion is satisfied. We controlled the depth of the tree to prevent over-fitting.
- *Support Vector Machine (SVM)*^105^ the algorithm generates a hyperplane to separate binary response variables by maximizing the total distances between the hyperplane and all data points. We assessed linear kernel, polynomial kernel, and radial basis kernel. The model with linear kernel is simple but had the best performance in our case.
- *Random Forest*^106^ random forest is an ensemble of multiple decision trees. The implementation we used takes the poll of predictions from individual trees as the final predictive result. Random forest is considered a complex model that possess strong predictive power.
- *Boosted Decision Trees*^107^ an algorithm that combines weak tree classifiers into a strong classifier using boosting approach. The implementation we applied is XGBoost^108^. The model is considered complex.

To be consistent with the process of assessing the PRS’s performances, we applied a fivefold cross validation procedure on the entire data to select the best hyper-parameters. One fold was used as the validation set and the other four folds were involved in parameter tuning and model constructing. We repeated the process until each one of the fivefolds was used as the validation set once. Then we constructed the final model on the entire data using the best parameters and calculated the feature importance. According to the official documentation from scikit-learn package^109^, the incorporated feature_importance function might inflate the importance of numerical variables using impurity-based measurement. Therefore, we adopted the suggested permutation_importance, though we did not notice significant differences between the two approaches. All the modeling procedures were conducted in Python (v3.7)^110^ with publicly available packages.

Note that we used the entire data as training set, without constructing a holdout test set. This step is taken to minimize the loss of power for statistical analysis considering our sample size. Since we carefully tuned hyper-parameters in the cross-validation process to protect against over-fitting, we think that the final model constructed on the entire data is generalizable.

### M10. Functional mapping and biological signature analysis

Functional annotation of the GWAS results was performed using the SNPnexus (v4.0) platform^111–114^ designed for annotation and interpretation of sets of SNPs derived from GWAS. SNPnexus maps SNPs in genomic loci to genes using positional mapping based on maximum distances between SNPs and genes. Biological function of the genes was annotated based on the Genetic Association Database and through literature searches. Biological signature analysis was carried out with the GENE2FUNC (v1.3.6) algorithm implemented in Functional Mapping and Annotation of Genome-Wide Association Studies (FUMA, v1.3.5)^115^. Of the 31 input genes, 21 were identified with a unique Entrez^116^ identification number and all genes with an Entrez identification number (35,142) were used as the background gene set for the hypergeometric test. The Molecular Signatures Database database^39,117^ (v7.0, August 2019) was used for the set of potential biological signatures. The Benjamini-Hochberg method was used as a correction for multiple testing with a maximum adjusted *p*-value of 0.05 for gene-set enrichment tests.

### Ethical approval

The Research Ethics Committees of Duke University and Peking University granted approval for the Protection of Human Subjects for the Chinese Longitudinal Healthy Longevity Survey, including collection of DNA samples used for the present study. The survey respondents who contributed their DNA samples gave informed consent before participation. All of the GWAS experiments and methods of analyses in the present study were performed in accordance with relevant guidelines and regulations. The Religious Order Study and the Rush Memory and Aging Project were approved by an Intuitional Review Board of Rush University Medical Center. All subjects signed an informed consent, an Anatomic Gift Act, and a repository consent to allow their biospecimens and data to be used for ancillary studies. Conduct of the studies was performed in accordance with relevant guidelines/regulations set forth by the Rush University Medical Center.

## Supplementary information

Supplementary Tables.

## Data Availability

The Chinese Longitudinal Healthy Longevity Survey (CLHLS) data are publicly available from National Archive of Computerized Data on Aging (NACDA). The genetic data can be obtained by requesting from the corresponding author Dr. Yi Zeng. Data on the ROSMAP studies can be requested at www.radc.rush.edu.
