# Supplementary material for: Genetic and non-genetic factors associated with the phenotype of exceptional longevity & normal cognition
Source: Sci Rep. 2020 Nov 5;10:19140. doi: 10.1038/s41598-020-75446-2 (PMC7645680; doi:10.1038/s41598-020-75446-2)
Supplement: Supplementary file 1 — Supplementary Tables. [file 41598_2020_75446_MOESM1_ESM.pdf]

Genetic and Non-Genetic Factors Associated with the Phenotype of Exceptional Longevity & Normal Cognition

Bin Han[1], Huashuai Chen[2,3], Yao Yao[7], Xiaomin Liu[5], Chao Nie[4,5], Junxia Min[6], Yi Zeng[2,7,*], and Michael W. Lutz[8,*]

[1]Department of Statistical Science, Duke University, Durham, NC, USA
[2]Center for the Study of Aging and Human Development, Medical School of Duke University, Durham, North Carolina
[3]Business School of Xiangtan University, Xiangtan, China
[4]BGI Education Center, University of Chinese Academy of Sciences, Shenzhen, China
[5]BGI–Shenzhen, Shenzhen, China
[6]The First Affiliated Hospital, Institute of Translational Medicine, School of Medicine, Zhejiang University, Hangzhou, China
[7]Center for Healthy Aging and Development Studies, National School of Development, Raissun Institute for Advanced Studies, Peking University, Beijing, China
[8]Department of Neurology, Medical Center, Duke University, Durham, North Carolina
[*]Correspondence and requests for materials should be addressed to M.L. (email: Michael.Lutz@duke.edu) or Y.Z. (email: zengyi@duke.edu)

Supplemental Material

SNP	Chr.	Position	Nearest Gene (+/- SNP Distance)	A1	A2	MAF	P	Odds Ratio	Lower-95CI	Upper-95CI
**Odds Ratio < 1**										
rs13198061	6	152,306,894	ESR1*	T	C	0.051	1.0×10^{-6}	0.49	0.36	0.65
rs939432	15	33,986,294	RYR3*	C	A	0.274	1.1×10^{-6}	0.70	0.61	0.81
rs954303	16	59,581,776	RNU4-58P (7606)	A	G	0.155	3.5×10^{-6}	0.66	0.56	0.79
rs1030695	4	130,318,150	RP11-419L4.1 (91973)	T	A	0.299	5.3×10^{-6}	0.72	0.63	0.83
rs4816332	21	30,201,706	N6AMT1 (42807)	C	T	0.400	7.5×10^{-6}	0.74	0.64	0.84
rs28617348	4	71,372,179	AMTN (12078)	C	T	0.448	9.4×10^{-6}	0.75	0.66	0.85
rs76299633	13	40,727,639	LINC00332 (28307)	G	A	0.119	1.4×10^{-5}	0.65	0.54	0.79
rs56368572	5	11,300,912	CTNND2*	T	C	0.094	1.4×10^{-5}	0.63	0.51	0.77
rs9404070	6	101,463,320	GRIK2 (383344)	G	A	0.415	1.5×10^{-5}	0.76	0.67	0.86
rs10500293	19	46,431,638	NOVA2 (5354)	G	A	0.440	1.7×10^{-5}	0.76	0.67	0.86
rs1293144	20	52,917,208	PFDN4 (72617)	T	G	0.371	1.8×10^{-5}	0.75	0.66	0.86
rs72627042	3	23,906,287	UBE2E1*	T	C	0.058	2.1×10^{-5}	0.54	0.41	0.72
rs1954882	18	48,290,668	MAPK4 (32474)	A	G	0.128	2.2×10^{-5}	0.67	0.56	0.81
rs62001981	15	25,279,909	RP11-701H24.10* & PWAR6*	T	C	0.196	2.9×10^{-5}	0.71	0.60	0.83
**Odds Ratio > 1**										
rs6726046	2	234,287,221	DGKD* & AC019221.4*	A	G	0.375	3.0×10^{-6}	1.36	1.20	1.55
rs935129	17	47,486,016	RP11-81K2.1* & PHB*	A	G	0.387	4.0×10^{-6}	1.36	1.20	1.55
rs13028996	2	234,246,225	SAG*	C	T	0.466	4.0×10^{-6}	1.36	1.19	1.55
rs6547617	2	85,655,402	SH2D6*	A	T	0.406	1.0×10^{-5}	1.36	1.19	1.55
rs2792251	1	164,541,977	PBX1*	G	A	0.136	1.4×10^{-5}	1.52	1.26	1.84
rs79669991	22	43,936,861	-	A	G	0.253	1.5×10^{-5}	1.39	1.20	1.62
rs2418761	10	107,295,345	RNU6-463P (822)	C	T	0.110	1.6×10^{-5}	1.58	1.28	1.94
rs7710849	5	82,220,225	RP11-78C3.1 (3287)	T	A	0.052	1.7×10^{-5}	2.02	1.47	2.78
rs10037430	5	180,569,007	OR2V2 (12936)	C	T	0.069	1.7×10^{-5}	1.91	1.42	2.56
rs8181601	11	44,759,309	TSPAN18*	G	C	0.107	1.7×10^{-5}	1.61	1.29	1.99
rs741171	16	6,652,854	RP11-420N3.2* & RBFOX1*	G	A	0.206	2.2×10^{-5}	1.42	1.21	1.66
rs4934715	10	35,364,992	CUL2*	T	G	0.306	2.3×10^{-5}	1.35	1.18	1.56
rs764967	21	36,299,665	RUNX1*	G	T	0.096	2.4×10^{-5}	1.61	1.29	2.00

Table 1. The table presents the information of clumped significant SNPs from GWAS with "education" as an additional covariate. To be consistent with our analysis, 3×10^{-5} was used as the p-value threshold. The nearest genes are either the genes that contain the variants (overlapping) or the nearest upstream/downstream gene to the variants. * indicates overlapping gene. Distances to the nearest upstream/downstream genes are listed in the parenthesis, measured in bp distance.

Feature	Cognitively Normal (N = 899)	Cognitively Impaired (N = 1257)	p-value
Age (years), mean (SD)	100.75 (3.77)	101.70 (3.46)	$< 1 \times 10^{-4}$ **
Education (years), mean (SD)	1.40 (3.12)	0.58 (1.84)	$< 1 \times 10^{-4}$ **
Sex			$< 1 \times 10^{-4}$ **
Male	306	227	
Female	593	1030	
Occupation			$< 1 \times 10^{-4}$ **
White-Collar	53	27	
Other	846	1230	
Marital Status			$< 1 \times 10^{-4}$ **
Single	832	1216	
Partnered	67	41	
Staple Food			$< 1 \times 10^{-4}$ **
Corn	25	42	
Rice	558	596	
Wheat	185	411	
Other	131	208	
Co-residence			$< 1 \times 10^{-4}$ **
Yes	729	1090	
No	170	167	
Fruit Intake			0.036 **
Yes	125	136	
No	774	1121	
Vegetable Intake			$< 1 \times 10^{-4}$ **
Yes	768	989	
No	131	268	
Current Smoker			0.014 **
Yes	87	84	
No	812	1173	
Former Smoker			$< 1 \times 10^{-4}$ **
Yes	201	179	
No	698	1078	
Current Drinker			0.008 **
Yes	118	118	
No	781	1139	
Former Drinker			9×10^{-4} **
Yes	204	212	
No	695	1045	
Exercise Currently			$< 1 \times 10^{-4}$ **
Yes	193	133	
No	706	1124	
Hypertension			$< 1 \times 10^{-4}$ **
Yes	193	181	
No	706	1076	
Diabetes			0.338
Yes	10	8	
No	889	1249	
Heart			0.367
Yes	83	101	
No	816	1156	
Cardiovascular Disease			0.035 **
Yes	34	74	
No	865	1183	
Respiratory			0.560
Yes	95	122	
No	804	1135	

Table 2. Summary statistics of non-genetic factors grouped on cognitive status. T-tests are performed to check whether there are significant differences in the mean values of continuous variables between the two cognitive groups. Chi-square tests of independence are conducted to check if the categorical variables are associated with the two cognitive groups. ** indicates that the p-value is less than 0.05.

	Cognitively Impaired at Any Age	Survival to Age 90+ years with Intact Cognition	Total	Difference Between the Phenotype Groups
N	276	579	855	
% Female	66%	76%	73%	p = 0.002
Age at baseline, years (mean (SD)))	78.0 (6.4)	83.3 (6.4)	81.6 (6.9)	p < 0.0001
Education years (mean (SD))	16.4 (3.9)	16.0 (3.4)	16.1 (3.6)	p = 0.19

Table 3. Demographic information of ROSMAP replication cohort.

Software	Version	Link
PLINK	1.9	http://www.cog-genomics.org/plink/1.9
IMPUTE	2.0	https://mathgen.stats.ox.ac.uk/impute/impute_v2.html
Kaviar	160204-Pubilc	http://db.systemsbiology.net/kaviar/
LDlink	4.1.0	https://ldlink.nci.nih.gov/
SNPnexus	4.0	https://www.snp-nexus.org/v4/
GENE2FUNC	1.3.6	https://fuma.ctglab.nl/
FUMA	1.3.5	https://fuma.ctglab.nl/
R	3.6.2	https://www.r-project.org/
Python	3.7	https://www.python.org/

Table 4. Versions of and links to the software mentioned in the manuscript
